# DAMPs and alarmin gene expression patterns in aging healthy and diseased mucosal tissues

**DOI:** 10.3389/froh.2023.1320083

**Published:** 2023-11-30

**Authors:** O. A. Gonzalez, S. S. Kirakodu, J. L. Ebersole

**Affiliations:** ^1^ Center for Oral Health Research, College of Dentistry, University of Kentucky, Lexington, KY, United States; ^2^ Department of Biomedical Sciences, School of Dental Medicine, University of Nevada Las Vegas, Las Vegas, NV, United States

**Keywords:** periodontitis, damage-associated molecular patterns, alarmins, microbiome, host response

## Abstract

**Introduction:**

Periodontitis is delineated by a dysbiotic microbiome at sites of lesions accompanied by a dysregulated persistent inflammatory response that undermines the integrity of the periodontium. The interplay of the altered microbial ecology and warning signals from host cells would be a critical feature for maintaining or re-establishing homeostasis in these tissues.

**Methods:**

This study used a nonhuman primate model (*Macaca mulatta*) with naturally-occurring periodontitis (*n* = 34) and experimental ligature-induced periodontitis (*n* = 36) to describe the features of gene expression for an array of damage-associate molecular patterns (DAMPs) or alarmins within the gingival tissues. The animals were age stratified into: ≤3 years (Young), 7–12 years (Adolescent), 12–15 years (Adult) and 17–23 years (Aged). Gingival tissue biopsies were examined via microarray. The analysis focused on 51 genes representative of the DAMPs/alarmins family of host cell warning factors and 18 genes associated with tissue destructive processed in the gingival tissues. Bacterial plaque samples were collected by curette sampling and 16S rRNA gene sequences used to describe the oral microbiome.

**Results:**

A subset of DAMPs/alarmins were expressed in healthy and naturally-occurring periodontitis tissues in the animals and suggested local effects on gingival tissues leading to altered levels of DAMPs/alarmins related to age and disease. Significant differences from adult healthy levels were most frequently observed in the young and adolescent animals with few representatives in this gene array altered in the healthy aged gingival tissues. Of the 51 target genes, only approximately ⅓ were altered by ≥1.5-fold in any of the age groups of animals during disease, with those increases observed during disease initiation. Distinctive positive and negative correlations were noted with the DAMP/alarmin gene levels and comparative expression changes of tissue destructive molecules during disease across the age groups. Finally, specific correlations of DAMP/alarmin genes and relative abundance of particular microbes were observed in health and resolution samples in younger animals, while increased correlations during disease in the older groups were noted.

**Conclusions:**

Thus, using this human-like preclinical model of induced periodontitis, we demonstrated the dynamics of the activation of the DAMP/alarmin warning system in the gingival tissues that showed some specific differences based on age.

## Introduction

Gingival tissues are constantly subjected to a range of stressors in the oral environment, many derived from the continuous challenge of the complex oral microbial ecology existing as biofilms juxtaposed to the tissues, including proteases (1–7), exotoxins (8–11), and endotoxins (12–19) among many other bacterial molecules. Moreover, these stimuli create a level of inflammation with associated inflammatory molecules derived from both resident cells and inflammatory cells that have emigrated from the vasculature in response to the local challenge (20–26). These activities often create a microenvironment for the cells that is increased in temperature (27–30), and pH (31–33), show altered electrolyte levels (34, 35), and elevated concentrations of reactive oxygen and nitrogen species (36–39) that can affect the physiology of the gingival tissue cells. Chronicity of this type of environment leads to destruction of both the soft tissues and alveolar bone, which result in the clinical measures of attachment loss and radiographic evidence of bone resorption that describe periodontitis.

It is also well recognized that the incidence and severity of periodontitis is increased with aging, leading to a “paradigm” of periodontitis as a frequent disease in the population reflecting long-term continuous noxious challenge to the tissues, which finally succumb to the destructive nature of the chronic stimuli (40–44). However, evidence provided over the last few decades has emphasized that the tissue destruction in periodontitis most likely is a manifestation of the chronic inflammatory response of the host to the complex microbial challenge that increases in quantity, as well as enabling the emergence of more pathogenic species to stimulate elevated levels of tissue destructive molecules (25, 45, 46). This view is also supported by ongoing genomic studies suggesting linkages between alterations in inflammatory gene expression and enhanced risk for development of periodontitis (47–52). Recent investigations have begun to emphasize that this chronic inflammation with tissue destruction is not a unidirectional process. Molecular studies have identified anti-inflammatory cytokines (53–59), pathways of inflammation resolving molecules (60–64), and molecules recognized as danger-associated molecular patterns (DAMPs) or alarmins (65–68) recognized by host cells to help minimize tissue damage and help to control destructive processes.

This report describes our studies using gingival tissues, as a representative mucosal tissue, derived from nonhuman primates (*Macaca mulatta*). We evaluated the expression of the DAMPs/alarmins that accompany healthy aging of these tissues. Finally, alterations in the expression of the DAMP/alarmin gene distribution and levels that reflect naturally-occurring and experimental periodontal disease across the lifespan and related to features of the oral microbiome in health and disease were delineated.

## Methods

### Nonhuman primate model and oral clinical evaluation

A population of rhesus monkeys (*Macaca mulatta*) housed at the Caribbean Primate Research Center (CPRC) at Sabana Seca, Puerto Rico, were used in this report and the previously published studies (69–72). “A protocol approved by the Institutional Animal Care and Use Committee (IACUC) of the University of Puerto Rico, enabled anesthetized animals to be examined for clinical measures of periodontal parameters including probing pocket depth (PPD), and bleeding on probing (BOP) as we have described previously (73). The nonhuman primates are typically fed a 20% protein, 5% fat, and 10% fiber commercial monkey diet (diet 8773, Teklad NIB primate diet modified: Harlan Teklad). The diet is supplemented with fruits and vegetables, and water is provided *ad libitum* in an enclosed corral setting” (74).

“For the naturally-occurring periodontitis model 34 animals were used (14 females and 20 males) reported in (75). Healthy animals (5–7/group) were distributed by age in 4 groups as follows: ≤3 years (young), 7–12 years (adolescent), 12–15 years (adult), and 18–22 years (aged). Only adult (*n* = 5) and aged animals (*n* = 6) with naturally-occurring periodontitis were used, since periodontitis does not occur in young animals. For this cohort, a clinical examination included probing pocket depth (PD) and bleeding on probing (BOP; 0–3 scale) (73). Clinical characterization for each group included: young and adolescent animals (mean PD < 2.0 mm, mean BOP < 0.5); adult healthy group (mean PD < 2.0 mm, mean BOP < 0.3); aged healthy group (mean PD < 3.0 mm, mean BOP < 1); adult periodontitis group (mean PD > 3.4 mm, mean BOP > 1.0); mean BOP < 1.0); and aged periodontitis group (mean PD > 3.4 mm, mean BOP > 1.5) described in (76).”

“For the longitudinal experimental ligature study, healthy animals were distributed by age into four groups of 9 animals/group (*n* = 36; 19 females and 17 males): ≤ 3 years (Young), 7–12 years (Adolescent), 12–15 years (Adult) and 17–23 years (Aged). A full mouth examination, excluding 3rd molars and canines, by a single investigator using a Maryland probe on the facial aspect of the teeth, e.g., 2 interproximal sites/tooth (mesio- and disto-buccal) was conducted. The healthy animals classified by a mean BOP of ≤1.0 and mean PPD of ≤3.0 were included. The animals then participated in a standard experimental ligature-induced periodontitis study (69, 72). Baseline (healthy) gingival tissues were biopsied from each animal from a single site prior to the experimental periodontitis procedure. Ligature-induced periodontal disease was then initiated whereby 2nd premolar and 1st and 2nd molar teeth in all 4 quadrants were ligated by tying 3–0 non-resorbable silk sutures around the cementoenamel junction of each tooth and using a periodontal probe to position the ligatures below the gingival margin. The methods were carried out in accordance with all relevant regulations for the use of nonhuman primates following ARRIVE guidelines (https://arriveguidelines.org/resources/author-checklists).”

“Further, clinical evaluation of ligated sites and gingival tissue samples were obtained at 0.5 (initiation), 1 (early progression), and 3 months (late progression). Determination of periodontal disease at the sampled site was documented by assessment of the presence of BOP and probing pocket depth of >4 mm, as we have described previously (69, 77). Then, ligatures were removed after sampling at 3 months and samples taken 2 months later (resolution) (69, 77). Since the removal of the ligature eliminates the local noxious mechanical challenge and decreases the microbial burden accumulating on the tooth, this process is similar to nonsurgical periodontal therapy in humans. Our previous reports have documented the significant increase in inflammation in all age groups of animals within the first two weeks of ligature placement identified as disease initiation. Additionally, we have shown that adult and aged animals demonstrated significantly greater destructive disease compared to young and adolescent animals in this experimental periodontitis model (78). These methods have been reported previously in (79).”

### Tissue sampling and gene expression microarray analysis

“A buccal gingival sample from either healthy or periodontitis-affected tissue from the premolar/molar maxillary region of each animal was taken at each time point using a standard gingivectomy technique (80), and maintained frozen in RNAlater solution. Total RNA was isolated from each gingival tissue using a standard procedure as we have described and tissue RNA samples submitted to the microarray core to assess RNA quality analyze the transcriptome using the GeneChip® Rhesus Macaque Genome Array (Affymetrix) (76, 81). Each animal provided 1 gingival biopsy for gene expression analysis at each time point. Thus, individual samples were used for the analytics. The genes explored in this study focused on 48 genes representative of this family of host biomolecules (Table 1). It must be recognized that the genes selected for the analysis represent those described in the literature as related to this biological warning process across the gingival cell population, and do not specifically address the cell types displaying these changes within the complex of the gingival tissues (74).”

**Table 1:** DAMP/alarmin genes analyzed.

Gene ID	Gene name	Gene ID	Gene name
ANXA1	Annexin A1	PCDH8	Protocadherin 8
APOA1	Apolipoprotein A1	PGLYRP1	Peptidoglycan recognition protein 1
APOA2	Apolipoprotein A2	PPIA	Peptidylprolyl isomerase A
APOB	Apolipoprotein B	PRDX2	Peroxiredoxin 2
APP	Amyloid beta precursor protein	PYCARD (ASC)	PYD And CARD domain containing
APOE	Apolipoprotein E	S100A8	S100 calcium binding protein A8
BGN	Biglycan	S100A9	S100 calcium binding protein A9
CALR	Calreticulin	S100A12	S100 calcium binding protein A12
CTSG	Cathepsin G	S100B	S100 calcium binding protein B
DCN	Decroin	SDC1	Syndecan 1
DDX58/RIGI	RNA sensor RIG-I	SFTPA1	Surfactant protein A1
DEFB103B/hBD3	Defensin beta 103B	SFTPD	Surfactant protein D
DEFB4/hBD2	Defensin beta 4A	SNAPIN	SNAP associated protein
ELANE	Neutrophil elastase	TNC	Tenascin C
FAM19A4/TAFA4	TAFA chemokine like family member 4	TSLP	Thymic stromal lymphopoietin
FGA	Fibrinogen alpha chain	VCAN	Versican
FGB	Fibrinogen beta chain	IL18	Interleukin 18
FGG	Fibrinogen gamma chain	PTGES2	Prostaglandin E synthase 2
FN1	Fibrinogen alpha chain	TNF	Tumor necrosis factor alpha
GNLY	Granulysin	CSF1	Colony stimulating factor 1
GPC1	Glypican 1	MMP1	Matrix metallopeptidase 1
HMGB1	High mobility group box 1	MMP3	Matrix metallopeptidase 3
HMGN1	High mobility group nucleosome binding domain 1	MMP8	Matrix metallopeptidase 8
HSP90AA1	Heat shock protein 90 alpha family class A member 1	MMP9	Matrix metallopeptidase 9
HSP90AB1	Heat shock protein 90 alpha family class B member 1	CTSK	Cathepsin K
HSP90B1	Heat shock protein 90 beta family member 1	RANK/TNFRSF11A	TNF receptor superfamily member 11a
HSPA1B/HSP70	Heat shock protein family A (Hsp70) member 1B	RANKL/TNFSF11	TNF superfamily member 11
HSPD1/HSP60	Heat shock protein family D (Hsp60) member 1	ALPL	Alkaline phosphatase, biomineralization associated
IFIH1	Interferon induced with helicase C domain 1	TRAF6	TNF receptor associated factor 6
IL16	Interleukin 16	CSPG4	Chondroitin sulfate proteoglycan 4
IL1A	Interleukin 1 alpha	NFATc1	Nuclear factor of activated T cells 1
IL33	Interleukin 33	WNT5a	Wnt family member 5A
LL-37/CAMP	Cathelicidin antimicrobial peptide	FOS	Fos proto-oncogene, AP-1 transcription factor subunit
LTF	Lactotransferrin	STAT1	Signal transducer and activator of transcription 1
OSCAR	Osteoclast associated Ig-like receptor		

We have also published previously regarding qPCR validation of the microarray results for 57 genes with altered expression related to age and/or disease, including SAA, S100A8, FN1, and IL1A from this targeted dataset (72, 75, 82–90). Generally, across this range of genes, the findings were consistent both directionally and in magnitude of differential expression.

## Microbiome and data analysis

**“**Bacterial plaque samples were collected by curette sampling from the subgingival pocket juxtaposed to the site providing the tissue sample at each time point. Only one sample was obtained at a time point from each animal, and the site was only sampled a single time. The plaque was suspended in sterile PBS and centrifuged for 10 min at 14,000 g to pellet the bacterial cells. DNA was extracted from the pelleted cells using a MagNA Pure LC DNA isolation kit III (Roche Applied Science) in MagNA Pure LC automated nucleic acid purification system (Roche Applied Science). DNA was quantified using a Nanodrop (ThermoFisher). The range of DNA obtained from the microbiome samples was 100 ng to 2.1 µg, with generally lower concentrations in baseline (healthy) samples. 5 ng of DNA was used in PCR and subsequently equimolar concentrations used for sequencing all samples as reported previously (74).”

“A Dual index paired-end sequencing approach for sequencing (91) using a MiSeq sequencer instrument was employed to determine the total composition of the oral microbiota from each sample as we have described previously (70, 92). First DNA from each sample was amplified using 16S universal primers, which amplifies 254 bp from V4 region of the 16S rRNA gene. Each primer consisted of the appropriate Illumina adapter, an 8-nt index sequence to identify each sample, a 10-nt pad sequence, 2-nt linker and the gene specific 16S forward or 16S reverse primer sequence. The resulting 254 bp 16S rRNA gene amplicons generated from each sample was purified and pooled in equimolar concentrations. These amplicon libraries were mixed with Illumina-generated PhiX control libraries and used for shotgun library construction using Nextera XT genomic library construction protocol. Sequencing of each DNA fragment was done using two sequence reads and two index reads by three sequence primers. The overall process of cluster generation, sequencing, image processing, de-multiplexing and quality score calculation was performed on the MiSeq instrument (Illumina).

Barcoded sequences from the MiSeq run were trimmed and aligned to similar 16S rRNA sequences from the SILVA version4 rRNA database (https://www.arb-silva.de/) using MOTHUR (v.1.38.0) pipeline (93). Artificial erroneous reads were corrected using the pre.cluster mothur command, and chimeric sequences were removed using the UCHIME (94) command in MOTHUR pipeline, followed by removal of non-archaeal/bacterial sequence removal. Sequences were clustered into phylotypes based on their sequence similarity and these binned phylotypes were assigned to their respective taxonomic classification using the Human Oral Microbiome Database (HOMD V13) (https://www.homd.org/ftp/16S_rRNA_refseq/HOMD_16S_rRNA_RefSeq/). We used the HOMD database to classify the OTUs. Since the study classified bacteria in the oral microbiome of rhesus monkeys by comparing to bacterial species present in humans (ie. HOMD) there were bacteria sequences identified at the genus level but unclassified by further species comparison since they are not sufficiently similar to existing entries present in the HOMD database. The cutoff percentage used in the MOTHUR software (classify.seqs command) for classifying sequences is 80%, which would leave some OTUs unclassified or classify only at the genus level. The raw data are deposited at BioProject ID PRJNA516659 through the NIH NCBI. The bacterial OTUs targeted in this report are provided in Table 2 and reported previously (70, 74).”

**Table 2:** Oral microbiome constituents evaluated.

OTU	Order	Genus/species
001	Fusobacteriales	*Fusobacterium*_unclassified
002	Spirochaetales	*Treponema*_unclassified
003	N/A	*Bacteria*_unclassified
004	Selenomonadales	*Selenomonas*_unclassified
005	Fusobacteriales	*Leptotrichia*_unclassified
006	Veillonellales	*Veillonella*_unclassified
007	Lactobacillales	*Streptococcus*_unclassified
008	Bacteroidales	*Prevotella*_unclassified
009	Synergistales	*Fretibacterium* sp. HMT_361
010	Veillonellales	*Veillonella parvula* HMT_161
011	Selenomonadales	*Selenomonas sputigena* HMT_151
012	Synergistales	*Fretibacterium*_unclassified
013	Bacteroidales	*Prevotella* sp. HMT_526
014	Clostridiales	*Catonella morbi* HMT_165
015	Flavobacteriales	*Capnocytophaga*_unclassified
017	Pasteurellales	*A. actinomycetemcomitans* HMT_531
018	Spirochaetales	*Treponema denticola* HMT_584
019	Pasteurellales	*Pasteurellaceae*_unclassified
020	Bacteroidales	*Bacteroidetes*_unclassified
021	Bacteroidales	*Porphyromonas gingivalis* HMT_619
022	Bacteroidales	*Porphyromonas* sp. HMT_279
023	Bacteroidales	*Prevotella enoeca* HMT_600
024	Lactobacillales	*Streptococcus* sp. HMT_058
025	Veillonellales	*Veillonellaceae*_[G-1]_unclassified
026	Spirochaetales	*Treponema maltophilum* HMT_664
027	Synergistales	*Fretibacterium fastidiosum* HMT_363
028	Absconditabacteria	SR1_[G-1] sp. HMT_345
030	Pasteurellales	*Chloroflexi*_[G-1] sp. HMT_439
032	Fusobacteriales	*Fusobacterium* sp. HMT_203
033	Bacteroidales	*Porphyromonas endodontalis* HMT_273
037	Clostridiales	*Eubacterium*_[XI][G-1] *infirmum* HMT_105
039	Bacteroidales	*Porphyromonadaceae*_unclassified
040	Bacteroidales	*Prevotella oris* HMT_311
041	Bacteroidales	*Prevotella* sp. HMT_820
045	Bacillales	*Gemella morbillorum* HMT_046
046	Clostridiales	*Peptostreptococcaceae*_[XI]_unclassified
048	Veillonellales	*Veillonellaceae*_[G-1] sp. HMT_155
050	Synergistales	*Pyramidobacter piscolens* HMT_357
053	Bacteroidales	*Prevotella* sp. HMT_317
056	Bacteroidales	*Prevotella* sp. HMT_304
058	Veillonellales	Veillonella dispar HMT_160
059	Bacteroidales	*Prevotella* sp. HMT_313
060	Spirochaetales	*Treponema socranskii* HMT_769
062	Bacteroidales	*Prevotella intermedia* HMT_643
063	Clostridiales	*Filifactor alocis* HMT_539
064	Lactobacillales	*Streptococcus parasanguinis*_II HMT_411
067	Clostridiales	*Peptostreptococcaceae*_[XIII]_unclassified
069	Pasteurellales	*Aggregatibacter*_unclassified
073	Fusobacteriales	*Leptotrichia* sp. HMT_223
074	Neisseriales	*Neisseria oralis* HMT_014
082	Bacteroidales	*Prevotella fusca* HMT_782
084	Desulfobacterales	*Desulfobulbus* sp. HMT_041
092	Veillonellales	*Megasphaera micronuciformis* HMT_122
093	Pasteurellales	*Haemophilus* sp. HMT_035
102	Bacteroidales	*Prevotella* sp. HMT_443
104	Spirochaetales	*Treponema* sp. HMT_246
116	Bacteroidales	*Prevotella denticola* HMT_291
170	Pseudomonadales	*Moraxella catarrhalis* HMT_833

## Statistical analysis

**“**The details of the gene expression intensities across the samples as reported previously (79) were estimated using the Robust Multi-array Average (RMA) algorithm with probe-level quintile normalization, as implemented in the Partek Genomics Suite software version 6.6 (Partek, St. Louis, MO). The data have been uploaded into GEO accession GSE180588 (https://www.ncbi.nlm.nih.gov/gds). Statistical differences of bacterial OTUs were determined with a *t*-test at least at *p* < 0.05. Correlation analyses were determined using a Pearson Correlation Coefficient with a *p*-value defined as *p* < 0.01 for the OTU-DAMP/alarmin gene comparisons.”

## Results

### DAMP/alarmin gene expression profiles in gingival tissues

**Figure 1:**
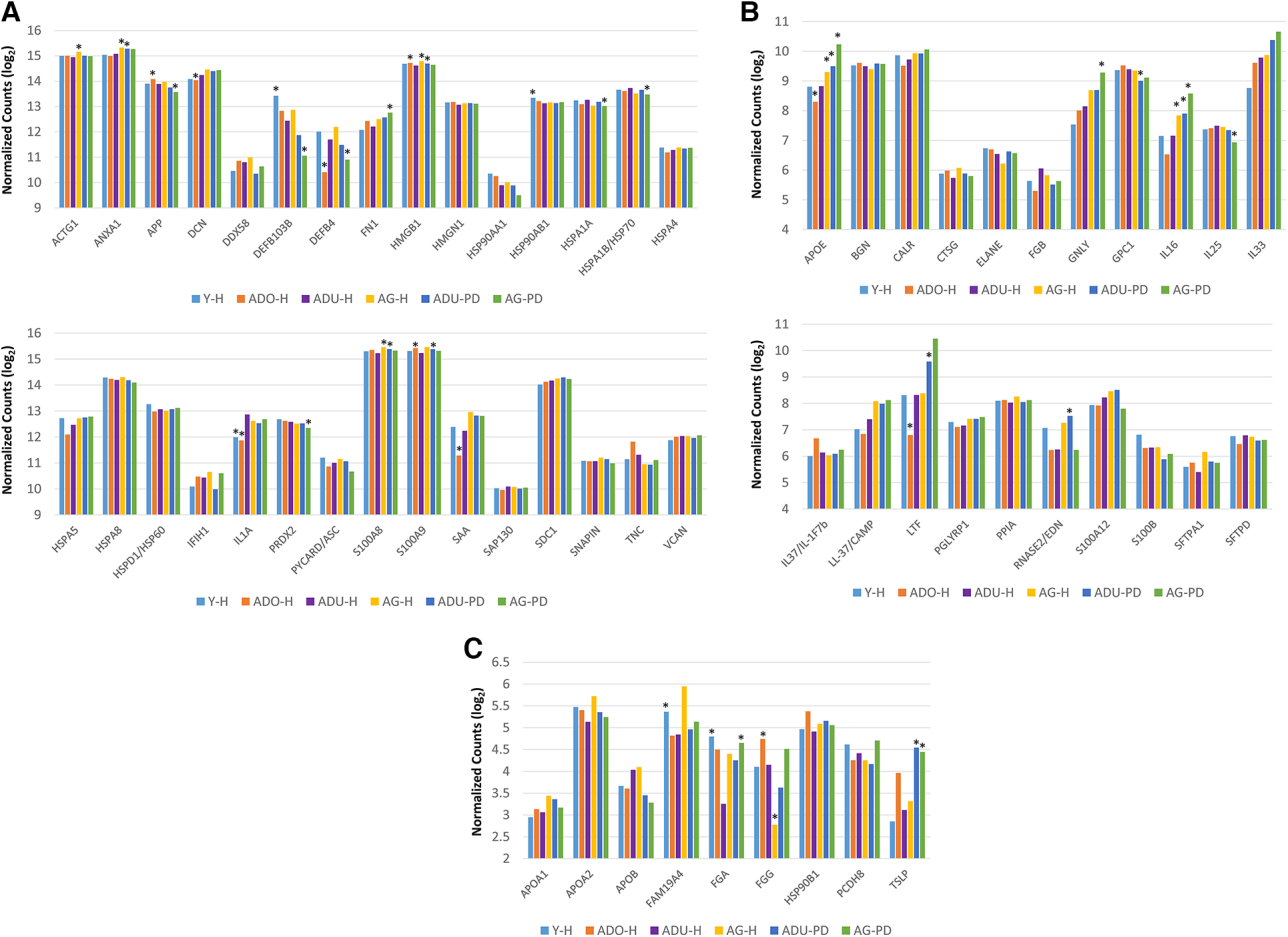
(**A**–**C**) normalized counts of DAMP/alarmin gene expression in gingival tissues from healthy animals of different ages (Y-H; ADO-H; ADU-H; AG-H) or with naturally-occurring periodontitis (ADU-PD; AG-PD). Asterisks (*) denote significantly different from ADU-H levels at *p* < 0.05.

The initial studies examined the levels of DAMPs/alarmins expressed in healthy and naturally-occurring periodontitis tissues in the animals (Figure 1). There results demonstrated some prominent genes expressed in healthy gingival tissues in all age groups. These included ACTG1, ANXA1, DCN, HMGB1, S100A8 and S100A9, APOE/ß2-GPI, IL33, and LTF. A number of these genes were also significantly increased in aging and periodontitis tissues (e.g., ANXA1, HMGB1, S100A8, S100A9, APOE/ß2-GPI, LTF, TSLP). Additionally, altered levels of DEF103B and IL1A were seen in younger healthy tissues. These results suggested local effects on gingival tissues leading to altered levels of DAMPs/alarmins related to age and naturally-occurring disease.

**Figure 2:**
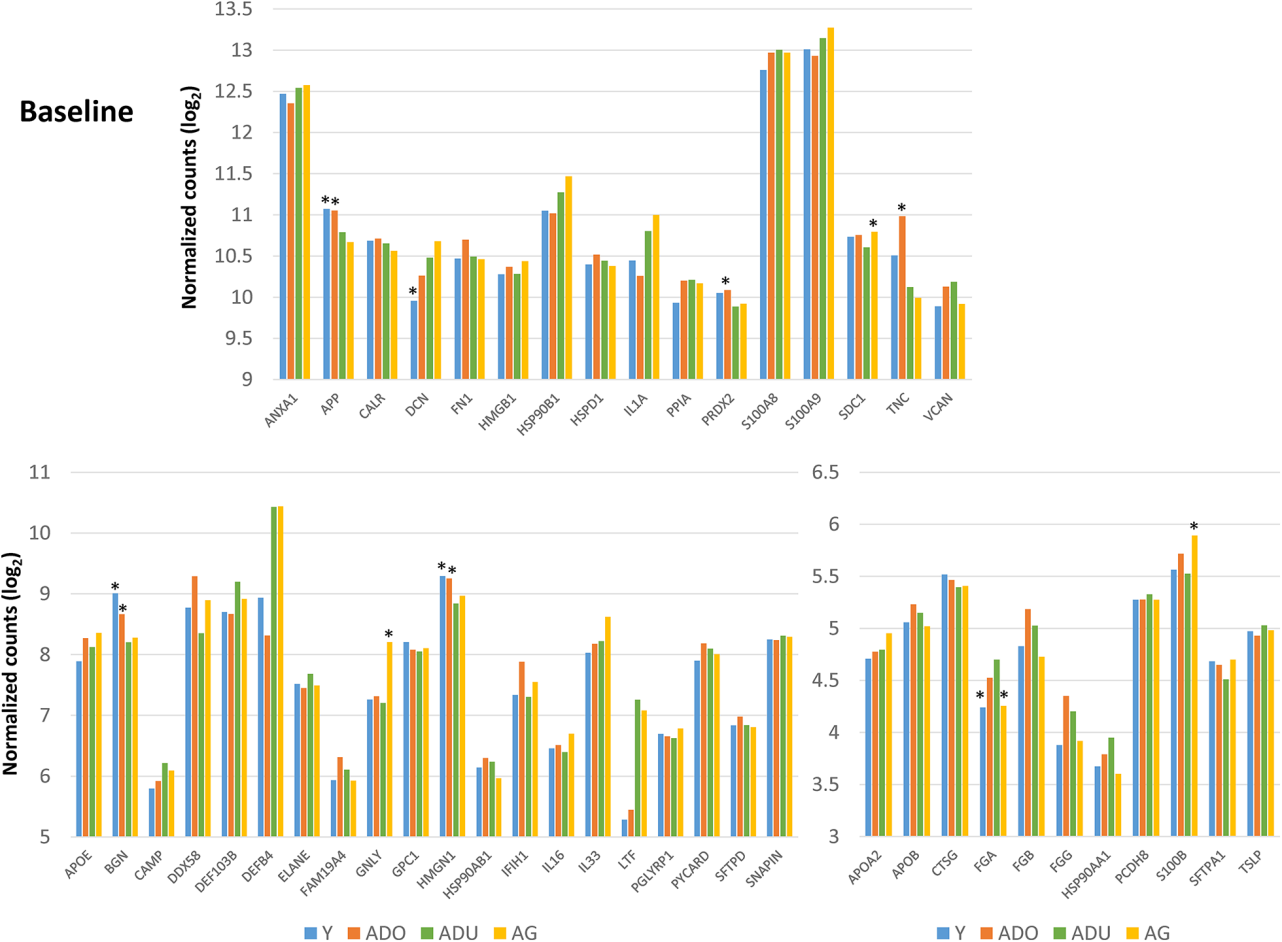
Normalized counts of DAMP/alarmin gene expression in healthy gingival tissues of different age groups at baseline of the experimental periodontitis model. Asterisks (*) denote significantly different from ADU-H levels at *p* < 0.05.

Figure 2 provides a summary of the DAMP/alarmin gene expression patterns in healthy gingival tissues for a subsequent prospective longitudinal study of experimental periodontitis. Significant differences from adult healthy levels were most frequently observed in the young and adolescent animals (e.g., APP, PRDX2, TNC, BGN, HMGN1, FGA). Few representative in this gene array were altered in the healthy aged gingival tissues.

**Figure 3:**
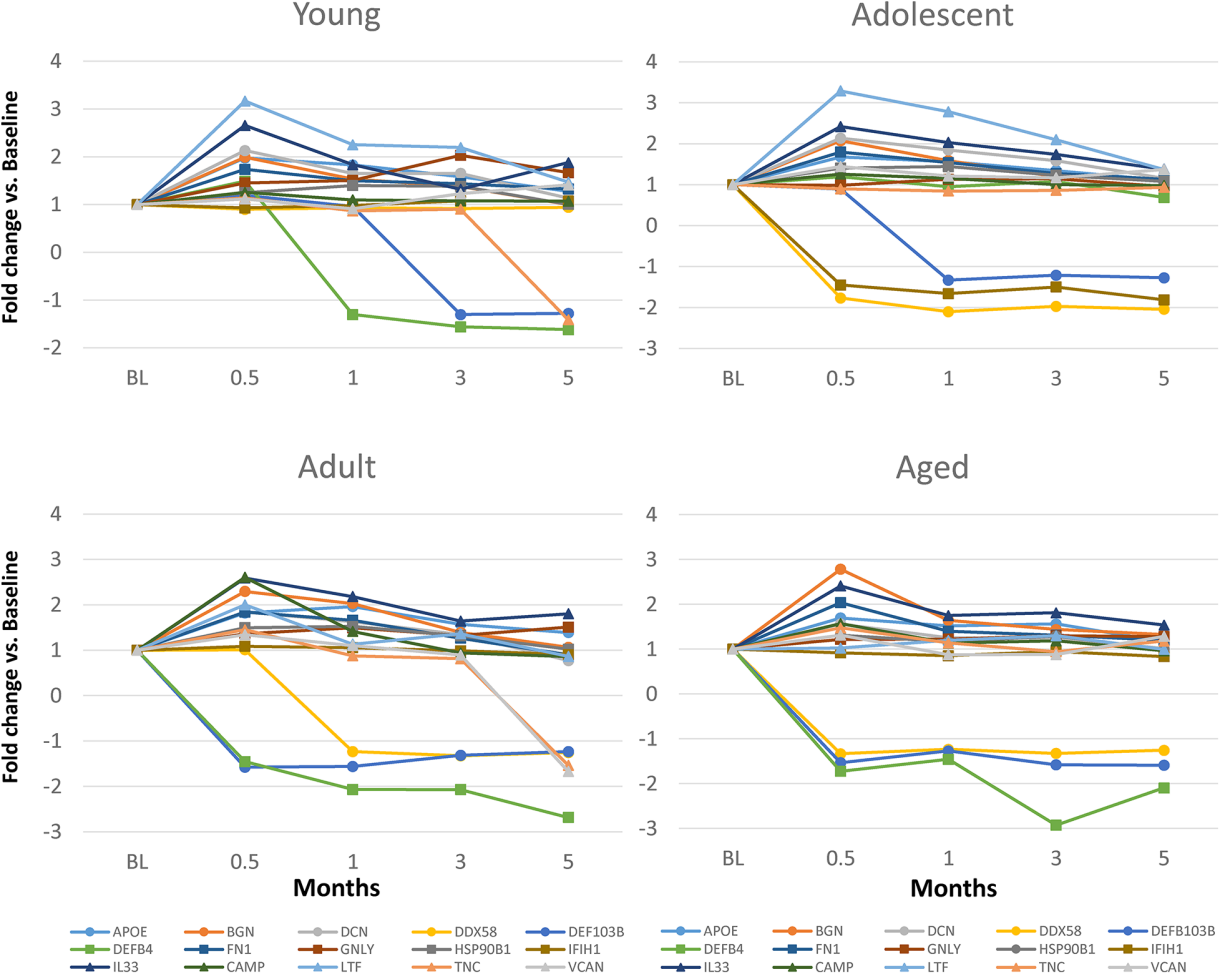
Fold changes from baseline (health) in selected DAMP/alarmin genes that showed a ≥1.5-fold difference at one or more time points in any of the age groups.

Figure 3 displays an overview of the changes in the gene expression during periodontitis and with resolution of the periodontal lesions. Of the 48 target genes, only 15 were altered by ≥1.5-fold in any of the age groups of animals. Generally, DEFB4, DEFB103B, DDX58 were decreased with disease, as was TNC in the young and adult animals. Increases in LTF, IL33, DCN, BGN, and FN1 were observed across the age groups, with increases observed during disease initiation (0.5 months) and maintaining these elevated levels throughout disease progression.

### Relationship of DAMP/alarmin expression to tissue destructive gene expression patterns

**Figure 4:**
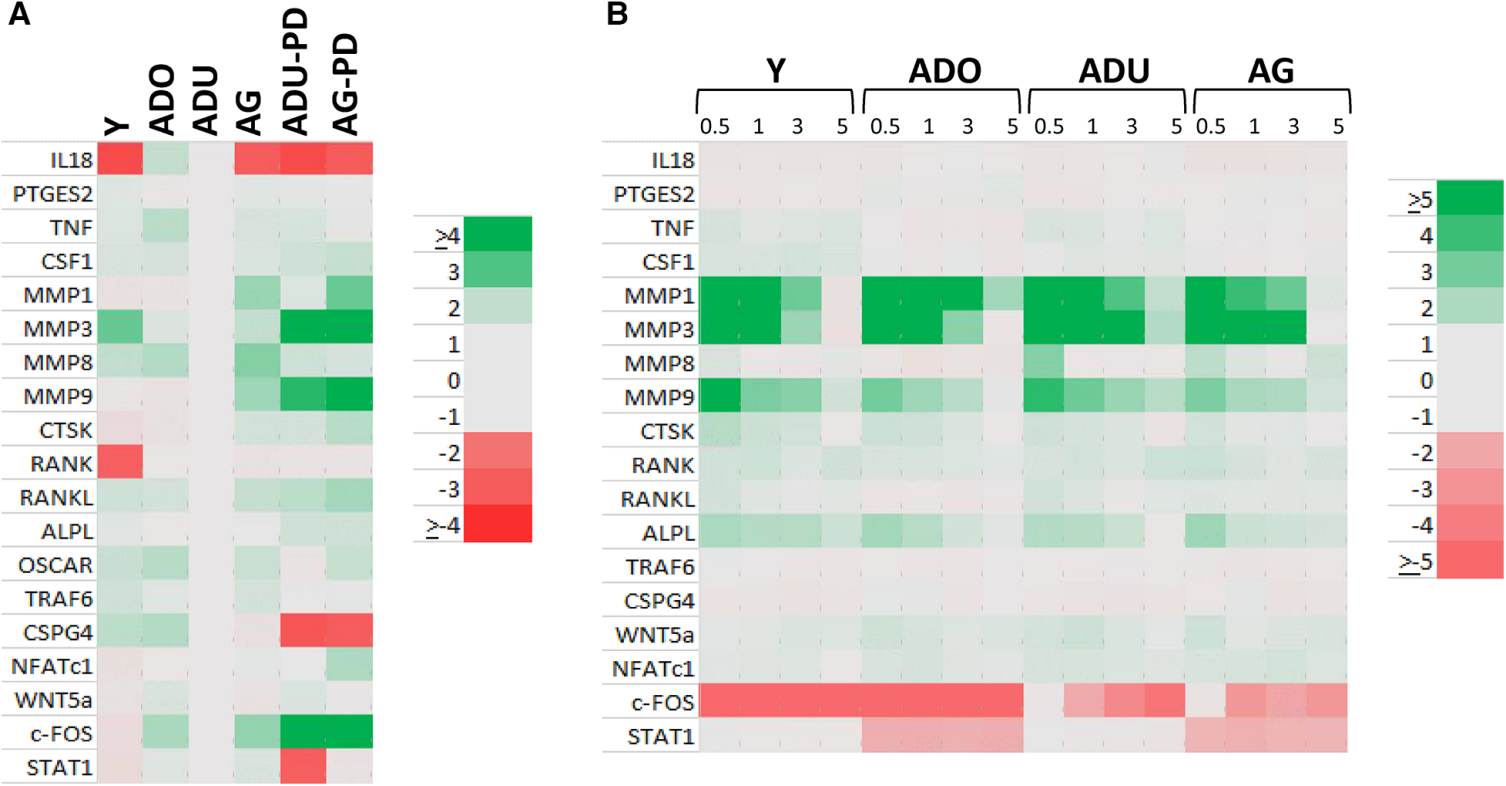
Heatmap of fold differences in normalized counts in (**A**) naturally-occurring disease model across age groups. Fold changes compared to ADU-H tissues. (**B**) Experimental periodontitis model across age groups and time points of disease (0.5, 1, 3) and resolution (5). Fold changes compared to baseline (healthy) tissues for each age group.

Changes in DAMP/alarmin levels in the gingival tissues can represent a component of the tissue alteration/destruction that it occurring during the formation of periodontal lesions. As such, Figure 4 summarizes the findings from a targeted set of genes that would be related to these tissue changes in disease. Figure 4A identifies increases in MMP1, MMP3, MMP9, CTSK, RANKL, NFATc1, and c-FOS with naturally-occurring disease, accompanied by decreases in IL18, CSPG4, and STAT1. Similar alterations were observed in the longitudinal experimental periodontitis model across all age groups with elevated MMP1, MMP3, MMP9, and ALPL. STAT1 was similarly decreased, albeit the levels of c-FOS differed between these disease models.

**Figure 5:**
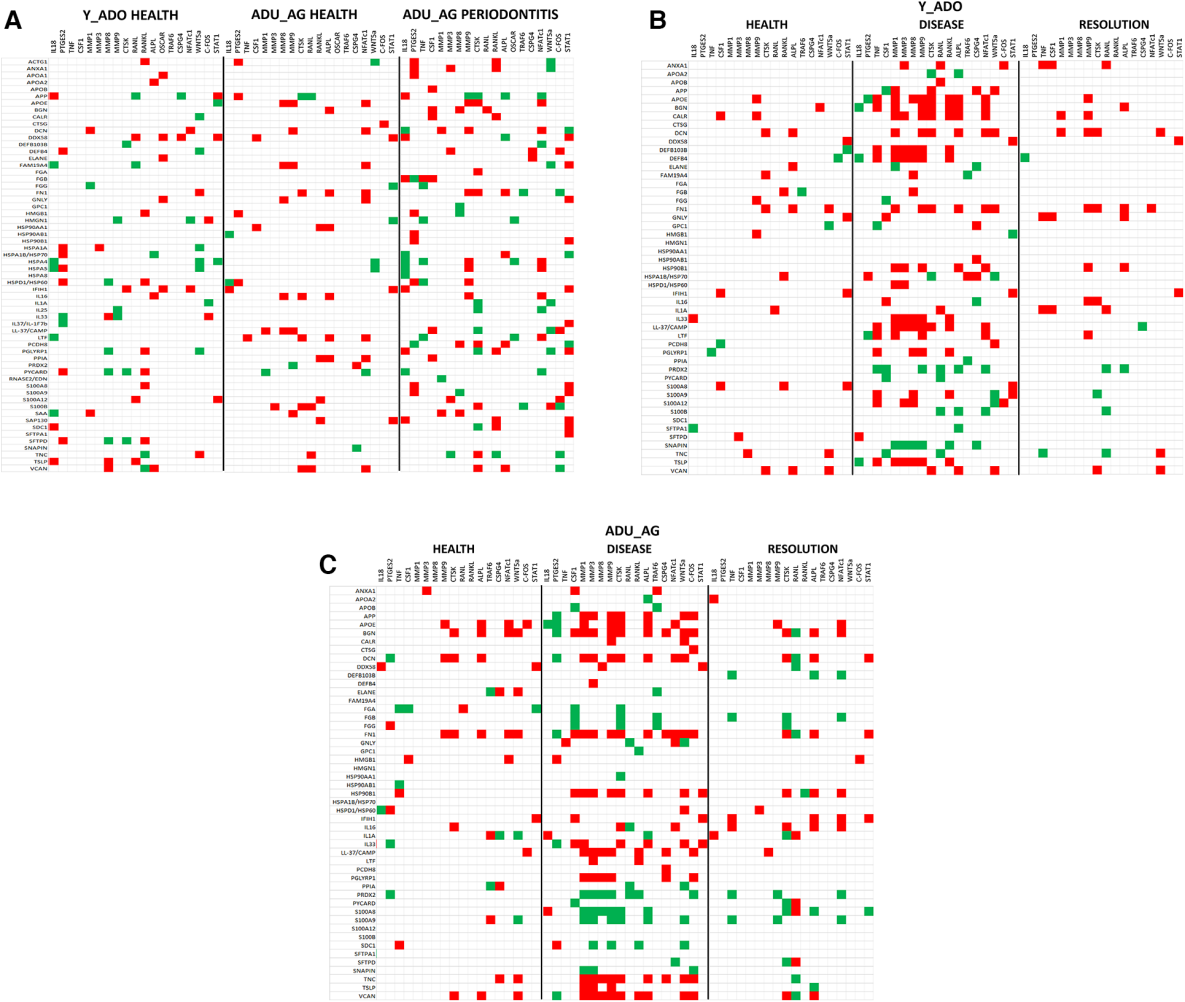
Summary of significant correlations between normalized counts of DAMP/alarmin genes and genes associated with inflammatory tissue destruction outcomes. Red denotes significant positive correlation and green denotes significant negative correlation at *p* < 0.01. (**A**) naturally-occurring disease model; (**B**) experimental periodontitis model in combined data from younger animals (young, adolescent); and (**C**) experimental periodontitis model in combined data from older animals (adult, aged).

The data analysis then focused in identifying patterns of the DAMP/alarmins that were related to potential tissue destructive changes (Figure 5). Figure 5A displays significant correlations of the DAMP/alarmin gene expression with the tissue destructive gene patterns in health compared with naturally-occurring disease. The results showed similar frequencies of positive and negative correlations in the young/adolescent samples. In contrast, in the adult/aged healthy samples nearly 5 times as many DAMP/alarmin genes were positively correlated with the tissue destructive gene levels. A similar skewed distribution was noted in the disease samples with approximately 50% more DAMP/alarmin genes positively correlated. Of interest was the distribution of DAMP/alarmin correlations across the samples with DDX58, FN1, IFIH1, IL16, VCAN, and HSPA4 showing a predilection for these relationships.

Figure 5B shows a similar analysis for the longitudinal experimental periodontitis model. With the young and adolescent animals, significant positive correlations dominated the relationships in both healthy and resolution samples. DCN and FN1 tended to show a higher frequency of these correlations in the two types of samples. Disease in this younger group of animals demonstrated a broad array of significant correlations with the genes related to tissue destructive activities. In this analysis, distinct patterns of positive correlations were specifically noted with soft tissue destruction processes, e.g., MMP, CTSK, as well as with bone biology related genes (e.g., RANKL, ALPL). Also of interest was that the DAMP/alarmins related to the correlations were also somewhat distinctive with APOE, BGN, CALR, DCN, DEFB103B, DEFB4, FN1, IL33, CAMP and TSLP. More limited negative correlations were observed, with PRDX2 and SNAPIN skewed towards this comparison.

Figure 5C provides a summary of similar outcomes for the adult and aged groups of animals. As with the younger groups, in health the positive correlations predominated with APOE, BGN, and FN1 showing more frequent correlations. With resolution in the older groups, the distribution of positive and negative correlations were more similar with rather limited patterns for any particular DAMP/alarmin gene. However, with disease there was a dramatic increase in significant correlations, including both positive and negative correlations. Similar to the younger groups, APP, APOE, BGN, FN1, CAMP, PGLYRP1, TNC, TSLP, and VCAN were all significantly related to MMPs and ALPL related to bone biology. Additionally, there was a large array of positive correlations with the various transcription factors. In contrast, PRDX2, S100A8 and S100A9 were negatively correlated with the MMPs and bone biology genes.

### Relationship of DAMP/alarmin expression to oral microbiome members

**Figure 6:**
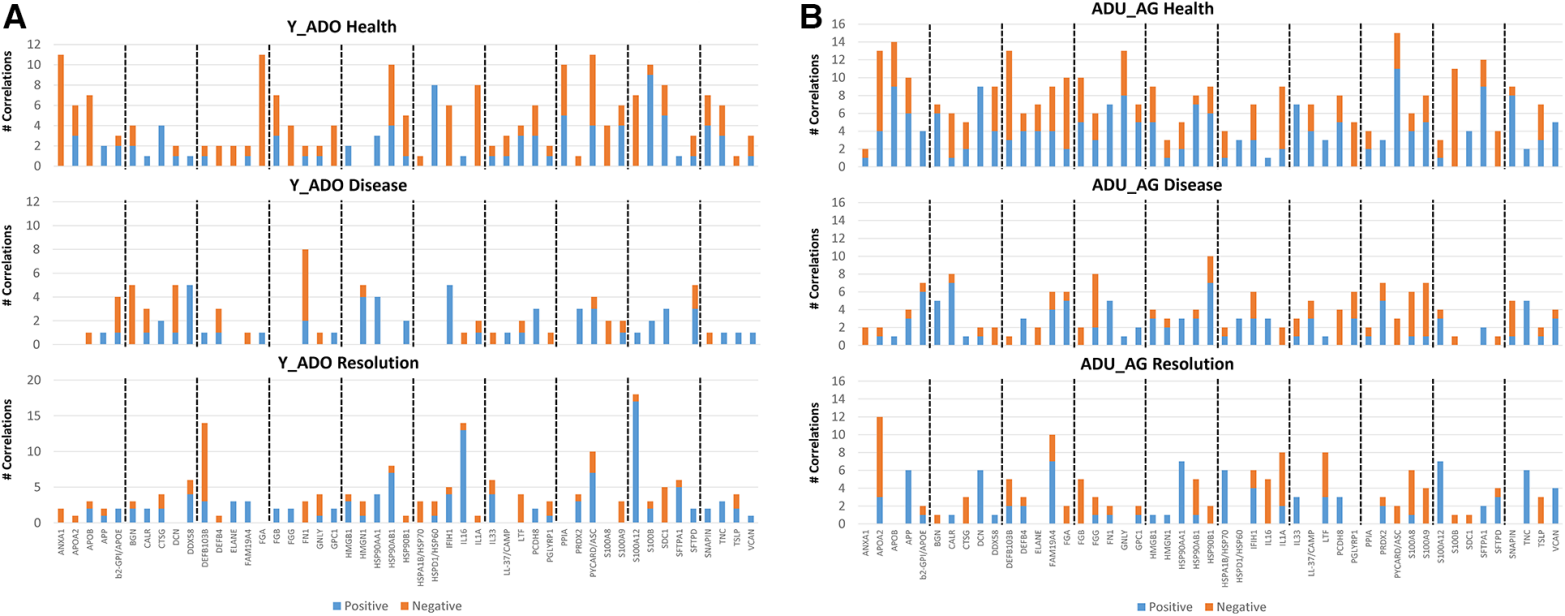
Comparison of the frequency of significant (*p* < 0.01) correlations between the normalized counts of DAMP/alarmin genes and the relative abundance of the overall taxa in the oral microbiome of (**A**) younger (young, adolescent) and (**B**) older (adult, aged) animals. Frequency of both significant positive and negative correlations are presented.

In Figure 6A, we evaluated the relationship between members of the oral microbiome in samples from sites in the experimental periodontitis model in health, progression periodontal lesions, and with resolution related to the expression of DAMP/alarmin genes. Forty-one OTUs represented the primary microbiome members in the young and adolescent samples. In healthy samples, the array of DAMP/alarmin genes showed significant correlations with the various OTUs particularly evident with ANXA1, FGA, HSP90AB1, HSPD1, IL1A, PPIA, PYCARD/ASC, S100B, and SDC1 in the healthy samples. The frequency of correlations decreased with disease, with only FN1 remaining highly correlated with various OTUs. Finally, in the resolution samples the microbial correlations of the DAMP/alarmin genes was limited with only DEFB103B, HSP90AB1, IL16, PYCARD/ASC, and S100A12 demonstrating an elevated frequency of correlations.

As in healthy samples from the younger animals, Figure 6B shows a greater frequency of correlations with the OTUs in healthy gingival tissues from the older groups of animals. These correlations were seen with APOA2, APOB, APP, DCN, DDX58, DEFB103B, FAM19A4, FGA, FGB, GNLY, HMGB1, HSP90AB1, HSP90B1, IL1A, PCDH8, PYCARD/ASC, S100B, SFTPA1, and SNAPIN. The frequency of correlations decreased in the disease samples with only CALR, FGG and HSP90B1 showing an elevated frequency. As in the young group, the resolution samples showed a low frequency of correlations, limited to APOA2, FAM19A4, IL1A, and LTF showing these correlations.

**Figure 7:**
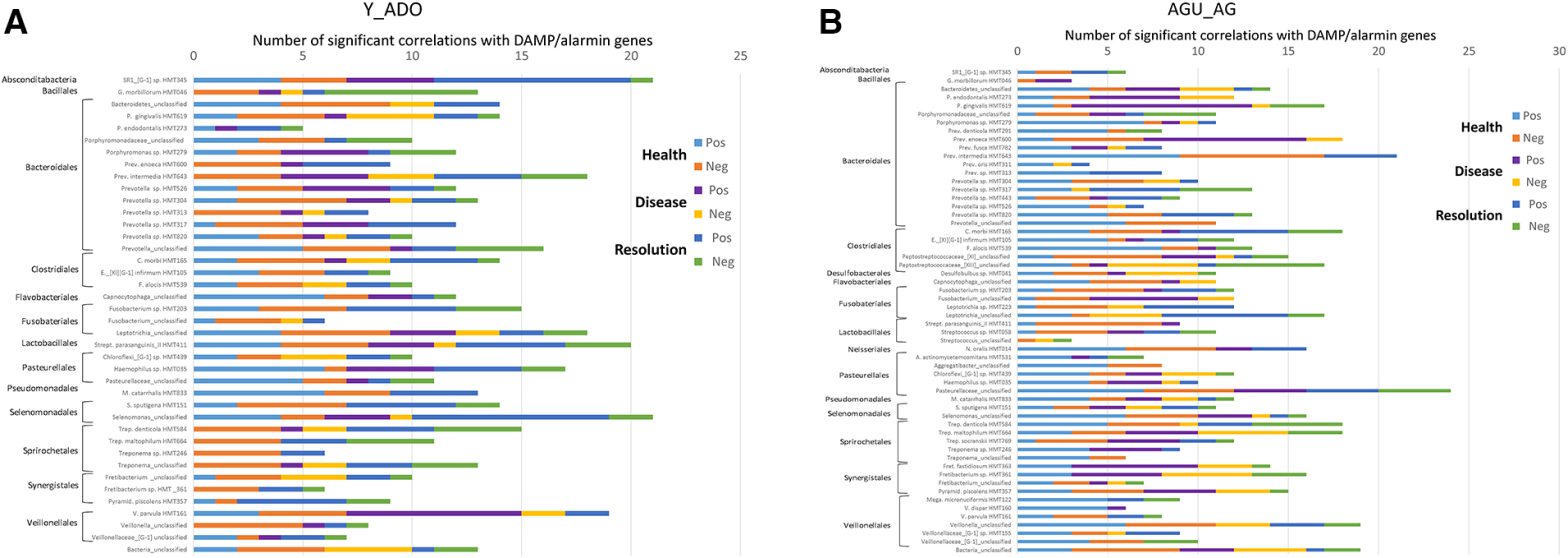
Frequency of significant (*p* < 0.01) correlations of the relative abundance of individual microbial taxa organized by order with the normalized counts of the array of DAMP/alarmin genes. Stacked bars summarize the number of positive and negative correlations in health, disease, and resolution samples from (**A**) younger (young, adolescent) and (**B**) older (adult, aged) animals.

The data was then evaluated to determine which member of the oral microbiome were most closely related to alterations in the DAMP/alarmin gene expression profiles in health, disease and resolution specimens. Figure 7A displays the frequency of significant correlations between an individual OTU and the array of 51 DAMP/alarmin genes. In the younger groups, SR1 HMT345, *Prevotella* intermedia HMT643, *Prevotella*_unclassified, *Fusobacterium* sp. HMT203, *Leptotrichia*_unclassified, *S. parasanguinis* HMT411, *Haemophilus* sp. HMT035, *Selenomonas*_unclassified, *T. denticola* HMT584, and *V. parvula* HMT161 were all highly correlated with the range of DAMP/alarmin genes. Generally, in this group of animals, the correlations were in the healthy and resolution samples, except with *Fretibacterium* sp. HMT361 and *V. parvula* HMT161, whereby the prevalence of correlations were in disease samples.

Figure 7B summarizes similar results from the adult and aged specimens. In these samples, *P. gingivalis* HMT619, *P. enoeca* HMT600, *P. intermedia* HMT643, *C. morbi* HMT165, *Peptostreptococcaceae* [XI]/[XII]_unclassified, *Leptotrichia*_unclassified, *N. oralis* HMT014, *Pasteurellaceae*_unclassified, *Selenmonas*_unclassified, *T. denticola* HMT584, *T. maltophilum* HMT664, *Fretibacterium* sp. HMT361, *P. piscolens* HMT357, and *Veillonella*_unclassified all showed a predominant frequency of correlations. Of these, *P. gingivalis, P. enoeca, Peptostreptococcaceae* [XII]_unclassified, *T. maltophilum, Fretibacterium,* and *P. piscolens* correlations were skewed towards disease samples.

## Discussion

At sites of local tissue destruction resulting from chronic inflammatory lesions, various triggers occur that presage the inflammatory responses. In periodontitis, the triggers include microbial stimulants derived from the complex oral bacterial biofilms transitioning from a symbiotic and homeostatic microbiome to a disease associated dystiotic community (45, 95). Additional stimulants are derived from disruption of host cell integrity and functions releasing an array of damage-associated molecular pattern biomolecules (DAMPs) that are considered alarmins for triggering protective/reparative host responses (65, 66, 68, 96–98). We have reported extensively on the methods for the use of this human-like disease model of naturally-occurring and experimental ligature-induced periodontitis (72, 76, 82, 87, 92). The model provided a large quantity of transcriptomic data from mucosal gingival tissues in health and disease. Querying the transcriptome provides the opportunity to target cellular biologic pathways that comprise the complex milieu of the periodontium and in this case, exploring features of these DAMPs and alarmin host molecules in the gingival tissues.

The study also evaluated the effect of age of the animals on the attributes of this host response system in health and disease. Across the array of DAMPs/alarmins, there were a limited number showing age effects and differences from healthy adult tissue levels. These were most frequently affected in the younger animals or in the aged and diseased samples. Also, in the longitudinal experimental periodontitis study, the distinctive DAMP/alarmin genes were commonly increased or decreased with disease initiation across the age groups. The characteristics of the decreased gene expression were targeted toward antimicrobial peptide production (DEPB4, DEFB103B), related to innate immune recognition processes especially viral related (DDX58/RIGI), and an extracellular matrix protein (TNC) important in guidance of cell migration. Those genes that were substantially increased following the initiation of disease featured antimicrobial molecules (LTF, CAMP), a chemoattractant for T cell maturation (IL33), and contributing to innate and adaptive immune responses in regulating immune suppressive activities (APOE). Additionally, the elevated gene expression profiles included genes related to fundamental cell functions that could trigger an alarmin response including DCN as a stimulator of autophagy and inflammation, BGN related to collagen fiber assembly and inflammation and innate immune responses, and FN1 focused on normal cell adhesion and migration capabilities.

The study was also designed to examine the expression of these DAMPs/alarmins as the linkage between bacterial triggers of inflammation and potential downstream host modulators of tissue integrity. Thus, we identified specific DAMP/alarmin genes that significantly correlated with an array of the bacteria detected in the microbiome samples from the juxtaposed healthy, disease, or resolution sites. The vice versa analysis also identified a set of the oral bacteria that appeared to be most related to altered expression of these DAMP/alarmin genes. A notable similarity occurred between samples from younger vs. older animals, with respect to the most frequent correlations were with the genes in healthy samples from the groups. A decreased frequency was observed across the samples during disease, and the lowest correlation prevalence was detected in the resolution samples. Here the differences showed in the younger animals that the correlations were predominantly in the healthy and resolution samples, while in the older animals the correlations were skewed towards disease samples.

The literature is replete with a description of innate immune system components specifically designed and expressed to detect that vast array of bacterial, viral, fungal, and other noxious stimuli by host cells (99–108). These detectors include both cell surface and intracellular components that evolved to combat specific components on extracellular and intracellular pathogens. In addition, many of these are directly involved in more physiologic functions to help maintain homeostasis with the autochthonous microbiota colonizing surfaces throughout the body. However, recognized as part of inflammation and innate immunity is the production/release of biomolecules that are specific warning signals to the host immune apparatus that cells in a particular location are undergoing stress and or mortal outcomes. These DAMPs or alarmins comprise a wide array of cell surface and intracellular components that reflect disruption of the integrity of cells. The host has developed a variety of detectors for these molecules and evolved to respond to reestablish homeostasis and stimulate resolution and healing (67, 96, 98, 100, 109, 110–112). Also clear is that these biomolecules are elevated in chronic inflammatory diseases, including periodontitis (##). These findings are generally derived from cross-sectional observational human studies, often comparing levels in tissues or fluids from healthy sites compared to established periodontal lesions or *in vitro* oral cell culture models.

Necroptosis is a method of programmed cell death that causes the release of DAMPs into the extracellular environment and thus transmit danger signals for inflammatory responses. This process can be induced by various periodontal pathogens and may play a role in this chronic inflammatory disease (113). Molecules contained in necrotic cell supernatants have been shown to stimulate inflammatory responses in both gingival epithelial cells and fibroblasts by activation of TLR3 (105). The presence and molecular mechanisms of release and detection of PAMPs and DAMPs has also been proposed to underpin the relationship of periodontitis and atherosclerosis, as well as presenting as potential therapeutic targets (65). Additional studies have presented the role of TLR and DAMP detection from damaged cells related to periodontal disease (114).

Information on the features of inflammasome activation that can result in pyroptosis and release of DAMPs in periodontitis is rather limited. However, this model of disease and engagement of the breadth of the host innate immune and inflammatory responses is likely important in this disease (115). More directly, DAMPs have been described in the oral cavity in periodontal disease, oral candidiasis, and Sjogren's syndrome. Not only do they likely intersect with exogenous signaling via pathogen-associated molecular patterns (PAMPs) but may aid in resolution of the oral fungal infection (66).

Various DAMPs/alarmins were found to be altered in the nonhuman primate tissues. IL-33, a member of the IL-1 family of cytokines, is a nuclear protein that is released from damaged cells as an alarmin (67). The cytokine was also elevated in GCF and plasma in generalized aggressive periodontitis patients compared to periodontally healthy or chronic periodontitis (116). Both IL-33 and IL-1α as alarmins were elevated in GCF and/or plasma in generalized aggressive periodontitis and in chronic periodontitis compared to healthy subjects (117). At the cellular level, IL-33 is overexpressed in gingival epithelial cells in chronic periodontitis and in a murine experimental periodontitis model with elevated expression of RANKL (118). Gingival fibroblasts have also been shown to release IL-33 induced by environmental TNFα levels (119).

HMGB1 is another major inflammatory mediator alarmin secreted by inflammatory cells. It interacts with TLRs to induce inflammatory cytokines. Paknejad et al. (111) showed that HMGB1 is released during inflammatory processes in periodontitis and likely promotes continued inflammation. Moreover, HMGB1 and the complement system provide important components of immune surveillance. Interplay between these pathways plays a role in chronic inflammatory diseases (120). This alarmin can be released by periodontal ligament cells and can modify macrophage differentiation and migration (121). There interplay between microbes and alarmins was suggested by a study showing that *F. nucleatum* was activates the NLRP3 inflammasome in gingival epithelial cells and causes the release of alarmins HMGB1 and ASC (PYCARD) from these cells (122).

Selected calcium-binding alarmins were also identified in this nonhuman primate disease model. S100A12, in the S100 subfamily of myeloid-related proteins acts as an alarmin to induce a pro-inflammatory innate immune response. It has been described to be elevated in various chronic inflammatory diseases. The molecule is produced by classical monocytes with cells from periodontitis patients producing higher levels compared to control subjects (123). More severe disease appeared to relate to the level of increase. S100A12 was also found to be increased in GCF and serum of CP patients and even more elevated in T2DM patients with periodontitis (124). S100A8, S100A9, S100A12, were all elevated in saliva from periodontitis and gingivitis patients compared to healthy controls (125) and salivary levels of S100A8, S100A9 and S100A12 were significantly related to clinical and radiographic signs of periodontitis (47). The S100A12 gene was a significant DEG in gingival tissues of periodontitis (126). Finally, GWAS studies of periodontitis found that S100A12 polymorphisms appear to influence periodontitis (127).

Finally, of the broad array of DAMP/alarmin molecules, SAA has been shown to be involved in the development of chronic inflammatory diseases. SAA is elevated in serum of periodontitis patients (128) and increased in inflamed gingival tissues where it triggers inflammatory cytokines via a TLR2 pathway (129). Obese patients with periodontitis were shown to have elevated levels of SAA in GCF (130). Additionally, in patients with coronary artery disease, inflamed gingival levels are correlated with systemic levels of SAA (Temelli 2018). Finally, Hirai et al. (110) showed SAA levels were positively associated with human periodontal inflammatory lesions. Mice lacking SAA also showed a decreased periodontal inflammatory infiltrate. The SAA levels appeared to be linked to TLR2/TLR4 functions in stimulating cellular responses.

Thus, differences in DAMP/alarmin levels have been linked to the clinical features of existing periodontal disease, albeit negligible data are available regarding the kinetics of the response related to disease process or the actual status of the particular affected site(s). Furthermore, minimal information is available regarding the biologic relationship between host factors involved in the tissue destructive events and the patterns of the DAMPs/alarmins in human lesions. Finally, while the alterations in more pathogenic oral microbiomes have been described, limited information is available concerning the potential linkage between changes in specific bacteria and the production of these biomarkers of cellular distress. Using this human-like preclinical model of induced periodontitis, we demonstrated the dynamics of the activation of the DAMP/alarmin warning system in the gingival tissues. The data indicated many similarities in the magnitude of these biomolecules across the lifespan, but did show some specific differences in young vs. older animals. The experimental design documented significant relationships between the elevated expression of DAMP/alarmin genes in the gingival tissues during the disease process and impacted by age. Further studies will be required to better understand how these relate to the biological triggers of the clinical disease features, as well as how they might be used as early harbingers of transition to disease, and/or therapeutic targets.

## Data Availability

The original contributions presented in the study are publicly available.The microbiological data can be found here: https://www.ncbi.nlm.nih.gov/, accession number PRJNA516659. The gene expression data have been uploaded into GEO accession GSE180588 (https://www.ncbi.nlm.nih.gov/gds).
